# Cost-effectiveness of a lifestyle intervention in high-risk individuals for diabetes in a low- and middle-income setting: Trial-based analysis of the Kerala Diabetes Prevention Program

**DOI:** 10.1186/s12916-020-01704-9

**Published:** 2020-09-04

**Authors:** Thirunavukkarasu Sathish, Brian Oldenburg, Kavumpurathu R. Thankappan, Pilvikki Absetz, Jonathan E. Shaw, Robyn J. Tapp, Paul Z. Zimmet, Sajitha Balachandran, Suman S. Shetty, Zahra Aziz, Ajay Mahal

**Affiliations:** 1 Melbourne School of Population and Global Health, University of Melbourne, Melbourne, Australia; 2 Population Health Research Institute, McMaster University, 237 Barton Street East, Hamilton, L8L 2X2 ON Canada; 3 WHO Collaborating Centre on Implementation Research for Prevention & Control of NCDs, University of Melbourne, Melbourne, Australia; 4 Achutha Menon Centre for Health Science Studies, Sree Chitra Tirunal Institute for Medical Sciences and Technology, Trivandrum, Kerala India; 5 Department of Public Health and Community Medicine, Central University of Kerala, Kasaragod, Kerala India; 6 Department of Public Health and Clinical Nutrition, University of Eastern Finland, Kuopio, Finland; 7 Faculty of Social Sciences, Tampere University, Tampere, Finland; 8 Baker Heart and Diabetes Institute, Melbourne, Australia; 9 School of Biomedical Engineering and Imaging Sciences, King’s College London, London, UK; 10 Centre for Intelligent Healthcare, Faculty of Health and Life Sciences, Coventry University, Coventry, Australia; 11 Central Clinical School, Monash University, Melbourne, UK; 12 Population Research Centre, University of Kerala, Trivandrum, Kerala India; 13 School of Psychological Sciences, Monash University, Melbourne, Kerala Australia

**Keywords:** Cost-effectiveness, Cost-utility, Diabetes, Lifestyle intervention, Prevention

## Abstract

**Background:**

Data on the cost-effectiveness of lifestyle-based diabetes prevention programs are mostly from high-income countries, which cannot be extrapolated to low- and middle-income countries**.** We performed a trial-based cost-effectiveness analysis of a lifestyle intervention targeted at preventing diabetes in India.

**Methods:**

The Kerala Diabetes Prevention Program was a cluster-randomized controlled trial of 1007 individuals conducted in 60 polling areas (electoral divisions) in Kerala state. Participants (30–60 years) were those with a high diabetes risk score and without diabetes on an oral glucose tolerance test. The intervention group received a 12-month peer-support lifestyle intervention involving 15 group sessions delivered in community settings by trained lay peer leaders. There were also linked community activities to sustain behavior change. The control group received a booklet on lifestyle change. Costs were estimated from the health system and societal perspectives, with 2018 as the reference year. Effectiveness was measured in terms of the number of diabetes cases prevented and quality-adjusted life years (QALYs). Three times India’s gross domestic product per capita (US$6108) was used as the cost-effectiveness threshold. The analyses were conducted with a 2-year time horizon. Costs and effects were discounted at 3% per annum. One-way and multi-way sensitivity analyses were performed.

**Results:**

Baseline characteristics were similar in the two study groups. Over 2 years, the intervention resulted in an incremental health system cost of US$2.0 (intervention group: US$303.6; control group: US$301.6), incremental societal cost of US$6.2 (intervention group: US$367.8; control group: US$361.5), absolute risk reduction of 2.1%, and incremental QALYs of 0.04 per person. From a health system perspective, the cost per diabetes case prevented was US$95.2, and the cost per QALY gained was US$50.0. From a societal perspective, the corresponding figures were US$295.1 and US$155.0. For the number of diabetes cases prevented, the probability for the intervention to be cost-effective was 84.0% and 83.1% from the health system and societal perspectives, respectively. The corresponding figures for QALY gained were 99.1% and 97.8%. The results were robust to discounting and sensitivity analyses.

**Conclusions:**

A community-based peer-support lifestyle intervention was cost-effective in individuals at high risk of developing diabetes in India over 2 years.

**Trial registration:**

The trial was registered with Australia and New Zealand Clinical Trials Registry (ACTRN12611000262909). Registered 10 March 2011.

## Background

Type 2 diabetes is a major cause of death and disability worldwide, with its health burden falling increasingly upon the low- and middle-income countries (LMICs) [1]. India has the second-largest number of people with diabetes in the world, with an estimated 77 million adults with the disease (21% of diabetes cases in LMICs) [1]. More worryingly, the prevalence of diabetes and the number of premature deaths due to diabetes are increasing rapidly in India and are likely to continue to impose a heavy economic burden on India’s healthcare system in the coming decades [2]. Identifying and implementing effective, cost-effective, and potentially scalable measures to control the diabetes epidemic is, therefore, a health policy priority for India.

Evidence from randomized controlled trials (RCTs) shows that lifestyle interventions for individuals at high risk of developing type 2 diabetes can reduce progression to diabetes [3], microvascular complications [4], and cardiovascular events [4], and improve cardiovascular risk factors [5, 6] and health-related quality of life (HRQoL) [7, 8]. Currently available evidence shows that lifestyle intervention is generally cost-effective in high-risk individuals for diabetes, but is based overwhelmingly on studies conducted in high-income countries [8]. Comparable studies from LMICs are scarce but are needed because interventions shown to be cost-effective in high-income countries are not generalizable to LMICs due to the differences in healthcare infrastructure, healthcare costs, and cost-effectiveness thresholds between these settings [9]. Furthermore, resources are much more limited in LMICs than in high-income countries, so cost-effective interventions in high-income countries may not be affordable in LMICs.

Only one previous study, the Indian Diabetes Prevention Programme (IDPP), has undertaken a cost-effectiveness analysis of lifestyle intervention among high-risk individuals for developing diabetes in India. The IDPP estimated a cost of US$1052 to prevent one case of diabetes over 3 years [10]. From an economic standpoint, the IDPP study had several limitations. First, it included only screening and intervention costs when assessing direct medical costs. By excluding healthcare use outside the immediate IDPP intervention components, the resulting cost estimates are likely to be either under- or overestimates of the incremental health system costs of the program, which are typically of greater policy interest. The cost-effectiveness of the IDPP from a societal point of view is also not known. Second, IDPP included only people with impaired glucose tolerance (IGT). While people with IGT are at high risk of developing diabetes, high-risk groups in the real world encompass a much broader subset of the population. These high-risk people without IGT but with other cardiovascular risk factors may outnumber people with IGT and require effective measures for diabetes risk reduction [11]. For example, in India, only about 4% of the adult population (20 years and older) have IGT, which is only 16% of people with prediabetes in India [12]. Finally, the IDPP study used a clinical endpoint (i.e., diabetes incidence) as an effectiveness measure and did not gather the information that could be used to generate quality-adjusted life years (QALYs) for cost-utility analysis. While lifestyle interventions can reduce diabetes incidence, they can also have broader health effects beyond glucose measures [5–7]. QALY captures the impact of an intervention on both the quantity and quality of life [13]. More importantly, the use of QALY enables a comparison of healthcare interventions across different medical conditions. This is pertinent, given India’s recent heavy investments in health technology assessments (HTAs) for promoting evidence-based choices to adopt health interventions by the health system [14].

We conducted a cluster-RCT of a community-based peer-support lifestyle intervention among individuals at high risk of developing type 2 diabetes in the Indian state of Kerala, the Kerala Diabetes Prevention Program (K-DPP) [6, 15]. After a mean follow-up of 2 years, the intervention resulted in a non-significant 12% relative risk reduction (relative risk 0.88, 95% CI 0.66 to 1.16) in diabetes incidence [6], significant 0.69% (95% CI 0.10–1.29%) absolute risk reduction in 10-year cardiovascular risk [16], and significant improvements in certain key cardiovascular risk factors, including physical activity, diet, tobacco use, alcohol use, and lipids, and HRQoL [6, 16, 17]. Resource constraints imply, however, that in addition to clinical effectiveness, any decision to adopt a health promotion program by a health system will also depend on its cost-effectiveness. We, therefore, report here the findings of the trial-based cost-effectiveness analysis of the K-DPP intervention from the health system and societal perspectives.

## Methods

### Study design, setting and participants

Details of the K-DPP study design have been previously published [15]. Briefly, 60 polling areas (electoral divisions) were randomly selected from the Neyyattinkara taluk (subdistrict) in the Trivandrum district of Kerala state. These polling areas were randomly assigned (1:1) to a control group or a lifestyle intervention group using a computer-generated randomization sequence by an independent person. Individuals (age 30–60 years) identified from the electoral roll of the selected polling areas were approached at their homes by trained field staff. Those with a history of diabetes or other major chronic conditions (e.g., cardiovascular disease, cancers), taking medications affecting glucose tolerance (e.g., steroids), illiterate in the local language, and pregnant women were excluded. Eligible individuals underwent a two-step screening procedure involving a diabetes risk score and a 2-h 75-g oral glucose tolerance test (OGTT) [18]. The Indian Diabetes Risk Score (IDRS) [19], which comprises age, family history of diabetes, physical activity, and waist circumference, was administered by trained staff. Those with an IDRS score of ≥ 60 were invited to attend clinics organized in local neighborhoods to undergo an OGTT. Those diagnosed with diabetes based on the American Diabetes Association (ADA) criteria (fasting plasma glucose [FPG] ≥ 7.0 mmol/l and/or 2-h plasma glucose [2-h PG] ≥ 11.1 mmol/l) [20] were excluded and referred to healthcare facilities for treatment and care. The remaining individuals who had a high diabetes risk score (IDRS ≥ 60) and did not have diabetes on the OGTT were recruited to the trial. Of these, 69% had prediabetes, and 31.0% had normal glucose levels based on the ADA criteria [20].

### Intervention

The development and cultural adaptation of the intervention program have been explained in detail elsewhere [21]. Briefly, the intervention was designed based on a needs assessment study [22] along with cultural adaptation from the components of evidence-based peer-support interventions tested in high-income countries, including Finland, Australia, and the USA [21]. The intervention consisted of 15 group sessions delivered over 12 months. All sessions were conducted in local neighborhoods in community buildings (e.g., schools, community halls) during Saturdays or Sundays at times that were convenient for participants. The K-DPP staff delivered introductory sessions (60–90 min/session) to introduce the group participants to the program and its mentoring style. Experts in the field of diabetes, nutrition, and physical activity delivered two half-day sessions focusing on prevention and management strategies for diabetes. Trained peer leaders (one male and one female per group), who were identified from within the group, delivered 12 sessions (60–90 min/session) at 1-month intervals on average. The peer group size varied from 10 to 23 participants. Content of the peer group sessions, and the selection and training of peer leaders have been reported elsewhere [6, 17]. The objectives of the lifestyle intervention were to increase physical activity, promote healthy eating habits and tobacco cessation, reduce alcohol consumption, maintain ideal bodyweight, and ensure adequate sleep. Peer leaders were given a handbook to assist them in running group sessions. Participants received resource materials including a handbook, a workbook, and a health education booklet to improve their knowledge on diabetes, risk factors, and prevention strategies and to monitor their progress towards lifestyle change. Bodyweight was measured during the peer group sessions. Additionally, participants were encouraged to have regular contact with their peer leaders outside the group sessions, and engage in community activities such as kitchen gardening, yoga training, and walking groups. Local resource persons (LRPs) identified for each group assisted the peer leaders in organizing the peer group sessions. Control group participants received a health education booklet on standard advice about lifestyle change.

### Costs

We estimated costs from the health system and societal perspectives, in accordance with the Consolidated Health Economic Evaluation Reporting Standards (CHEERS) statement [23] and cost-effectiveness analysis guidelines [24, 25]. Research costs were excluded from the analyses.

#### Health system costs

To estimate health system costs, we considered only direct medical costs, which included screening costs, intervention costs, and healthcare utilization costs.

##### Screening and intervention costs

Most of our screening costs were driven by the need to identify high-risk individuals for the trial in a short period of time through home visits and community-based clinics [18, 26]. From this perspective, many of the screening costs were considered to be research costs. In a non-research setting in India, health workers screen and identify high-risk individuals for diabetes as part of their ongoing clinical care [27]. In line with this, as well as based on previous diabetes prevention studies [10, 28], we considered only IDRS and OGTT costs for this cost-effectiveness analysis. The unit cost of these tests was obtained from the study accounts register (see Additional file 1: Table S1). A detailed description of the methods for estimating the intervention costs has been published previously [6]. Apart from peer leaders (60), the staff involved in delivering the group sessions were intervention manager (1), intervention assistant (1), LRPs (30), and experts (4). Although a time-use survey was not undertaken, a detailed log was maintained for the staff time spent on various intervention activities, and this time was valued based on staff remuneration rates. Cost of printing resource materials and operations (training, phone calls, travel, rent for intervention venues, and overheads) was based on the expenditure on these items. The unit cost of intervention components was obtained from the study accounts register (see Additional file 1: Table S1).

##### Healthcare utilization costs

Information on healthcare use (i.e., outpatient visits, inpatient days, and medication use) from public and private facilities was collected annually based on self-reports or from participants’ prescriptions, medical records, and bills, if available. In Kerala, the majority of outpatient (63.5%) and inpatient care (66%) are sought from private facilities [29]. Private health expenditure in Kerala, primarily household out-of-pocket payments, accounts for 73.4% of total health expenditure [30]. Thus, ignoring the utilization of services from private health facilities (and associated expenses) will significantly underestimate the true health system costs. The unit cost of outpatient visits and inpatient days at public and private health facilities was based on the World Health Organization (WHO) CHOICE (CHOosing Interventions that are Cost-Effective) estimates for India (see Additional file 1: Table S1) [31]. The cost of medications was obtained through participants’ self-reports or from prescriptions, medical records, and bills, if available.

#### Societal costs

To estimate societal costs, we considered direct medical costs, direct non-medical costs, and indirect costs. Direct non-medical costs included expenses incurred by participants for transport, food, and accommodation while seeking healthcare (self-reported), and for the time spent traveling to and attending group sessions. The intervention participants (including peer leaders) spent, on average, 30 min of travel time and 60 min in attending one group session. These times were valued at the minimum hourly wage of an unskilled worker employed in the agricultural sector in India [32]. Indirect costs were calculated by assuming that each inpatient stay resulted in a loss of 9 h of paid work, and each outpatient visit resulted in a loss of half a day (4.5 h) of paid work [32]. These lost days of productivity were valued at the minimum hourly wage of an unskilled worker employed in the agricultural sector in India for all participants [32]. This wage rate was used primarily for two key reasons: (1) the study population is predominantly rural, and the main source of employment for most people is the agricultural sector, which is primarily unskilled work; (2) if homemakers, retired, and unemployed wish to work at all, then they have to balance work with their home responsibilities, and unskilled work is probably what is most readily available to them. All costs in Indian Rupees (INR) were inflated for the year 2018 using the Consumer Price Index for India [33] and were converted to US dollars using an exchange rate of US$1 = INR68.4 [33].

### Effects

We assessed effectiveness in terms of the number of diabetes cases prevented and of QALYs, as recommended by the CHEERS statement [23] and cost-effectiveness analysis guidelines [24, 25]. The number needed to treat (NNT) to prevent one case of diabetes was estimated as the inverse of absolute risk reduction, i.e., the difference in diabetes incidence between the study groups [34]. There was no statistically significant difference in the absolute risk reduction in diabetes incidence (2.1% (95% CI − 2.9 to 7.1%, *p* = 0.405)) at 2 years. Our trial was likely underpowered to detect a significant difference, given the inclusion of participants with normal plasma glucose (31% by the ADA criteria; 65.9% by the WHO criteria) [35], and the short follow-up. Based on the memorable adage “absence of evidence is not evidence of absence” [36], Briggs and O’Brien argue that analysts should focus on estimating cost-effectiveness based on the joint analysis of costs and effects rather than relying on a stepwise approach that conditions the analysis on the statistical significance of cost or effect differences between treatment groups in the first step [37]. This is now recommended in many cost-effectiveness guidelines [24, 25] and is increasingly seen in the health economics literature [38–40].

QALYs were estimated based on the utility values derived from the 36-item Short Form (SF-36) health survey [41]. SF-36 was administered at baseline, 1 year, and 2 years. The SF-36 is divided into eight scales (physical functioning, role limitation—physical, role limitation—emotional, bodily pain, general health, mental health, social functioning, and vitality) and two domains (physical component summary and mental component summary). Scores for each of the scales and domains range from 0 to 100, with higher scores indicating a better quality of life. The SF-36 data were converted into a six-dimensional health state called the Short Form 6 Dimension (SF-6D), whose score ranges between 0.29 (worse health) and 1.00 (full health) [42]. The SF-6D data were converted to QALYs using the area under the curve method [43].

### Statistical analysis

This cost-effectiveness analysis was conducted using standard methods of trial-based economic evaluation [25]. The analyses were performed using the intention-to-treat approach. Missing values for healthcare use, diabetes incidence, and QALYs were imputed using the last observation carried forward method based on the missing completely at random assumption [44]. Generalized estimating equations (GEE) using log-binomial models with an exchangeable working correlation structure and robust standard errors to account for clustering by polling areas were used to estimate the absolute risk reduction in diabetes incidence between study groups. Generalized linear models (GLM) with gamma family and log link components were used to estimate the incremental costs and QALYs [45]. Standard errors for incremental QALYs were estimated by accounting for clustering by polling areas [46]. QALY models were adjusted for the baseline utility values (SF-6D). The incremental cost-effectiveness ratio (ICER) was estimated by multiplying the incremental costs with the NNT for diabetes incidence or by dividing the incremental costs with the incremental QALYs.

We estimated uncertainty in the estimates of costs and effects by non-parametric bootstrapping method, which was conducted by resampling without replenishment from the original dataset [47]. In each of the 1000 iterations, GEE (for diabetes incidence) or GLM (for costs and QALYs) were run, and the incremental costs, absolute risk reduction in diabetes incidence, and incremental QALYs were estimated. These estimates are presented visually using a cost-effectiveness plane and a cost-effectiveness acceptability curve. The cost-effectiveness plane shows uncertainty in the costs and effects in four quadrants, namely southeast (intervention is less costly and more effective than the control group), northeast (intervention is more costly and more effective than the control group), southwest (intervention is less costly and less effective than the control group), and northwest (intervention is more costly and less effective than the control group). The cost-effectiveness acceptability curve shows the probability for the intervention to be cost-effective, compared to the control group, at various willingness to pay (WTP) thresholds. WTP was based on the gross domestic product (GDP) per capita, with an intervention deemed to be cost-effective if the ICER is less than three times GDP per capita, as recommended by the WHO-CHOICE [31]. According to the International Monetary Fund, the GDP per capita for India in 2018 was US$2036 [33]. Analyses were performed with a 2-year time horizon (average length of K-DPP follow-up). The discount rate for costs and effects for the base-case scenario was 3% per annum, as recommended by the Panel on Cost-Effectiveness in Health and Medicine [24].

Additionally, we conducted the following one-way and multi-way sensitivity analyses. We allowed the cost of group sessions (the key driver of intervention costs) and effects to vary by a range of plausible values (i.e., ± 10, ± 20, and ± 30%) in a non-research setting, based on assumptions made in previous lifestyle-based diabetes prevention programs [28, 48]. This was done in three ways: the cost was allowed to vary, but the effects were kept fixed; the effects were allowed to vary, but the cost was kept fixed; and both costs and effects were allowed to vary. Since a 3% discount rate is based on guidelines developed for high-income countries, we discounted costs and effects using different rates (5% and 10% per annum) that are recommended for LMICs, including India [49]. Finally, we conducted multiple imputation (MI) analysis, accounting for clustering by polling areas [50, 51] to assess the impact of missing data on costs, diabetes incidence, and QALYs on the main results. MI was performed using chained equations with 10 imputations, and GEE and GLM models were run on each of the 10 imputed datasets, and the results were combined using Rubin’s rule [52]. Analyses were performed using Stata software (version 15.0; StataCorp, TX, USA) and Microsoft Office Excel 2018 (Microsoft Corporation, Redmond, WA, USA).

## Results

A total of 1007 participants (500 in the intervention group and 507 in the control group) were recruited into the trial. All 60 clusters and 964 (95.7%) participants (96.4% in the intervention group and 95.1% in the control group) were followed up at 2 years. The baseline characteristics of clusters and participants were similar in the two study groups (see Additional file 1: Table S2) [35].

### Costs

Table 1 summarizes the per capita cost over the 2-year trial period by the study group. Total intervention costs amounted to US$12,096 in the intervention group (US$24.2 per participant) and US$389 in the control group (US$0.8 per participant). Over 2 years, the outpatient and medication costs were lower in the intervention group by ~ US$12, compared to the control group. From a health system perspective, the mean cost per participant was US$303.6 in the intervention group and US$301.6 in the control group, resulting in an incremental cost of US$2.0. The total non-medical costs were higher in the intervention group compared to the control group by US$5.7. From a societal perspective, the mean cost per participant was US$367.8 in the intervention group and US$361.5 in the control group, resulting in an incremental cost of US$6.2.

**Table 1:** Average per capita cost by study group over 2 years

	Control group (*N* = 507)	Intervention group (*N* = 500)
**Direct medical costs**		
*Screening*		
IDRS	0.1	0.1
OGTT	4.1	4.1
*Intervention*		
Group sessions		
Introductory sessions	0	3.5
DPES	0	2.1
Peer group sessions	0	7.1
Training of peer leaders and LRPs	0	2.8
Resource materials	0.8	5.3
Community activities	0	0
Overheads	0	3.3
Subtotal	0.8	24.2
*Healthcare use*		
Outpatient visits	62.3	50.0
Inpatient days	47.5	50.5
Medications	186.8	174.8
Subtotal	296.6	275.2
Total cost from a health system perspective	301.6	303.6
**Direct non-medical costs**		
Transport, food, and accommodation costs while seeking healthcare	12.7	12.2
Travel time to attend group sessions	0	2.1
Time spent attending group sessions	0	4.1
Subtotal	12.7	18.4
**Indirect costs** (productivity loss due to illness)	47.2	45.8
Total cost from a societal perspective	361.5	367.8

*IDRS* Indian Diabetes Risk Score, *OGTT* oral glucose tolerance test, *DPES* diabetes prevention education sessions, *LRP* local resource person. Costs are expressed in 2018 US$

### Effectiveness

At 2 years, the absolute risk reduction in diabetes incidence was 2.1% (95% CI − 2.9 to 7.1%, *p* = 0.405), resulting in an NNT of 47.6. The average QALYs gained were consistently higher in the intervention group compared to the control group in both the first (0.81 vs. 0.79) and second years (0.84 vs. 0.81). Overall, during the 2-year trial period, participants in the intervention group accrued more QALYs than the control participants (1.65 vs. 1.61) with a mean difference of 0.04 (95% CI 0.02 to 0.07, *p* = 0.002).

### Cost-effectiveness

**Fig. 1:**
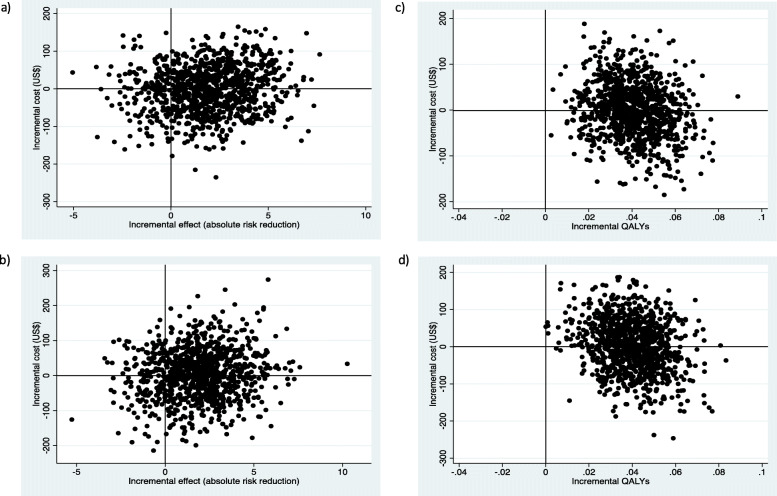
Cost-effectiveness plane. Absolute risk reduction in diabetes incidence: health system perspective (**a**) and societal perspective (**b**). QALY gained: health system perspective (**c**) and societal perspective (**d**). QALY, quality-adjusted life year

**Fig. 2:**
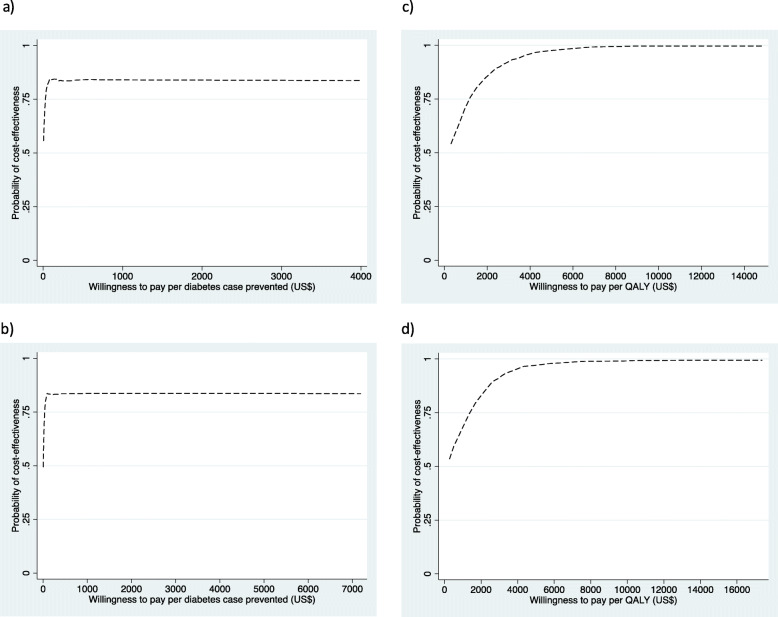
Cost-effectiveness acceptability curve. Diabetes cases prevented: health system perspective (**a**) and societal perspective (**b**). QALY gained: health system perspective (**c**) and societal perspective (**d**). QALY, quality-adjusted life year

Table 2 shows the incremental cost-effectiveness of lifestyle intervention over 2 years. From a health system perspective and compared with the control group, the lifestyle intervention costs US$95.2 per diabetes case prevented and US$50.0 per QALY gained. From a societal perspective and compared with the control group, the lifestyle intervention costs US$295.1 per diabetes case prevented and US$155.0 per QALY gained. A 3% discount rate per annum did not alter the ICERs appreciably. Figure 1 shows the cost-effectiveness plane. For diabetes cases prevented, from a health system perspective, the lifestyle intervention was associated with higher effects and lower costs in 41.6% of bootstrap estimates (southeast quadrant), and with higher effects and higher costs in 42.2% of bootstrap estimates (northeast quadrant). From a societal perspective, the corresponding figures were 39.8% and 43.3%. For QALYs gained, from a health system perspective, the lifestyle intervention resulted in higher effects and lower costs in 49.9% of bootstrap estimates (southeast quadrant), and in higher effects and higher costs in the remaining 50.1% of bootstrap estimates (northeast quadrant). From a societal perspective, the corresponding figures were 47.9% and 52.1%. Figure 2 shows the cost-effectiveness acceptability curve. For diabetes cases prevented, the probability for the intervention to be cost-effective was 84.0% and 83.1% at a WTP of < US$6108 (three times India’s GDP per capita) from the health system and societal perspectives, respectively. For QALYs gained, the corresponding figures were 99.1% and 97.8%.

**Table 2:** Incremental cost-effectiveness of the lifestyle intervention versus control group over 2 years

	Cost per diabetes case prevented		Cost per QALY gained	
	Health system perspective	Societal perspective	Health system perspective	Societal perspective
**Base-case analysis**				
Without discounting	95.2	295.1	50.0	155.0
With discounting (3% per annum)	95.1	292.5	49.0	150.8
**Sensitivity analyses**				
*Cost of group sessions only*				
10% increase	157.1	357.0	82.5	187.5
20% increase	219.0	414.1	115.0	217.5
30% increase	276.1	476.0	145.0	250.0
10% decrease	38.1	238.0	20.0	125.0
20% decrease	Dominated*	176.1	Dominated*	92.5
30% decrease	Dominated*	114.2	Dominated*	60.0
*Intervention effectiveness only*				
10% increase	87.0	269.7	45.5	140.9
20% increase	80.0	248.0	41.7	129.2
30% increase	74.0	229.4	38.5	119.2
10% decrease	105.2	326.1	55.6	172.2
20% decrease	117.6	364.6	62.5	193.8
30% decrease	133.4	413.5	71.4	221.4
*Both cost of group sessions and intervention effectiveness*				
10% increase	143.6	326.3	75.0	170.5
20% increase	184.0	348.0	95.8	181.3
30% increase	214.6	370.0	111.5	192.3
10% decrease	42.1	263.0	22.2	138.9
20% decrease	Dominated*	217.6	Dominated*	115.6
30% decrease	Dominated*	160.1	Dominated*	85.7
*Discounting*				
5% per annum	116.8	292.5	60.5	151.5
10% per annum	169.4	292.7	88.4	152.7
*MI analysis*				
Lifestyle intervention vs. control group	99.2	302.1	56.0	157.5

*QALY* quality-adjusted life year, *MI* multiple imputation

*Lifestyle intervention was less costly and more effective than the control group

### Sensitivity analyses

Table 2 shows the results of sensitivity analyses. ICERs were more sensitive to changes in the cost of group sessions than to the changes in effects. For example, from a health system perspective, a 10% increase in the cost of group sessions increased the ICER from US$95.2 to US$157.1, whereas the same increase in absolute risk reduction in diabetes incidence resulted in an ICER of US$87.0. For diabetes cases prevented, from a health system perspective, changes in the costs and effects resulted in ICERs ranging between dominance (lifestyle intervention was less costly and more effective than the control group) and US$276.1. From a societal perspective, the corresponding figures were US$114.2 and US$476.0. For QALYs gained, from a health system perspective, changes in the costs and effects resulted in ICERs ranging between dominance and US$145.0. From a societal perspective, the corresponding figures were US$60.0 and US$250.0. Discounting of costs and effects at 5% or 10% per annum and MI analysis did not alter the main results appreciably.

## Discussion

This trial-based cost-effectiveness analysis showed that the incremental cost of the lifestyle intervention was US$95.2 and US$295.1 per diabetes case prevented, and US$50.0 and US$155.0 per QALY gained, from the health system and societal perspectives, respectively, over 2 years. The uncertainty analysis indicates that more than 83% (for diabetes cases prevented) and 100% (for QALYs gained) of bootstrap estimates were cost-effective (<US$6108) from both the perspectives. In addition, the probability for the intervention to be cost-effective was more than 83% for diabetes cases prevented and 97% for QALYs gained from both the perspectives. Discounting costs and effects at a rate of 3% per annum did not alter the results appreciably. In all case scenarios in the sensitivity analyses for both the effects, the ICERs remained below the cost-effectiveness threshold (i.e., US$6108).

In our study, the difference in intervention costs between study groups (US$23.4) was accompanied by lower (roughly equal) outpatient, medication, and indirect costs in the intervention group, resulting in a negligible cost difference between study groups from the health system and societal perspectives. This could be due to the positive intervention effects on healthy lifestyle behaviors including physical activity, diet, tobacco use, and alcohol use [6, 17]. In LMICs, so far, two trial-based analyses [10, 53] and a modeling study [54] have evaluated the cost-effectiveness of lifestyle-related diabetes prevention programs. In the IDPP study, the ICER was US$1052 per case of diabetes prevented in those with IGT over 3 years from a health system perspective [10]. In the DMagic trial from Bangladesh, the ICER for a community mobilization intervention in people with intermediate hyperglycemia was INT$6518 per case of diabetes prevented (or INT$2551 per disability-adjusted life years averted) over 2 years from a health system perspective [53]. Additionally, a modeling study from China estimated that among those with IGT, diet, and physical activity interventions were cost-saving over 40 years from a societal perspective [54]. ICERs in our study cannot be directly compared with ICERs from these other LMIC studies, given the differences in cost perspectives, population groups, and effectiveness measures used. However, our study ICERs suggest higher levels of cost-effectiveness than those reported in high-income countries even after adjusting for differences in income per capita [8]. For example, in the US Diabetes Prevention Program (DPP), the cost per QALY gained was US$31,512 over 3 years from a health system perspective [28], indicating an ICER that is 630 times as high as our estimate (US$50 per QALY), whereas the US GDP per capita is 31 times as high as India’s GDP per capita [33].

While the NNT to prevent one case of diabetes in our study (47.6) was larger than that reported in the IDPP (6.4) [10] and US DPP (6.9) [28], the cost per case of diabetes prevented was much lower in our study. The IDPP and US DPP recruited people with IGT, whereas our study included participants based on a high diabetes risk score, with the majority having isolated impaired fasting glucose (i-IFG) (57.5%) [35]. Currently available evidence suggests that lifestyle interventions that are effective in reducing diabetes risk in people with IGT are not effective in those with i-IFG [11]. We estimated QALYs based on the preference-based health utility values derived from the SF-36 [41]. A review of 23 studies found that SF-36 provided the smallest change in health utility values, which generally translate to less favorable ICERs, compared to EuroQol 5D and other HRQoL scales [55]. Therefore, the QALY gain seen in our trial is likely to be a conservative estimate. Nevertheless, the magnitude of QALY gain (0.04) observed in our study is similar to or higher than that seen with many other lifestyle-related diabetes prevention interventions that are more intense or expensive [8]. For example, in the US DPP, the difference in QALYs between the lifestyle intervention and placebo groups was 0.02 at 2 years [28]. In the Let's Prevent Diabetes cluster-RCT from the UK, the QALY gain with the lifestyle intervention was 0.04  over 2 years [38].

Our study has several strengths. To our knowledge, this is the first study from an LMIC setting to provide comprehensive evidence on the cost-effectiveness of lifestyle intervention in high-risk individuals for type 2 diabetes from both the health system and societal perspectives. The K-DPP trial was conducted in the Indian state of Kerala, which has the highest prevalence of diabetes in India with a high burden of several cardiovascular risk factors [12, 56–58]. The epidemiological transition that is currently occurring in Kerala is indicative of what will happen in other states in India in the coming years, as well as in many other LMICs [59]. Therefore, evaluating the cost-effectiveness of appropriate lifestyle intervention strategies in this setting will enable proactive policymaking in other states of India as well as other LMICs. More importantly, in our study, we included high-risk individuals across all categories of glucose tolerance [35]. This, along with the pragmatic nature of intervention delivery, advances the generalizability of our study results to a broader high-risk population of India. While Kerala’s literacy rate, health indicators, and health system are comparatively better than most Indian states, we believe that our intervention model can be adapted to other Indian states and possibly to other South Asian countries. Factors likely to facilitate its application in other Indian settings include the relatively low cost of the intervention, the large presence of civil society organizations and self-help groups in rural Indian settings [60], and the recent government efforts to revitalize primary care and prevention strategies for non-communicable diseases [27]. Cost and QALY data were available for the vast majority of study participants (95.1%). We estimated uncertainty in the costs and effects, unlike many other trial-based cost-effectiveness analyses of diabetes prevention programs [8]. Finally, the main results were robust to several types of sensitivity analyses.

Our study has some limitations. These include, the relatively short follow-up of 2 years, which did not permit an assessment of the long-term cost-effectiveness of the intervention. However, by adopting a 2-year time horizon, we likely underestimated the effects, with the health benefits of a lifestyle intervention generally occurring over the longer term [61]. Another key limitation is that information on healthcare use was mainly self-reported and, therefore, susceptible to recall bias. However, this bias was reduced to some extent, as approximately 40% of healthcare use data was obtained from participants’ medical records, prescriptions, and bills. Moreover, in the Indian context, people tend to have good recall as a large proportion of total health expenditure (India’s average, 58.7% vs. 67% in Kerala) is paid out-of-pocket at the point of service delivery [30]. It is likely that homemakers, retired, and unemployed people have a lower opportunity cost of time than working individuals and have a greater likelihood of attending the peer group sessions and implementing steps to lower the risk of diabetes. On the other hand, time costs saved in the future from delayed or prevented diabetes would be much higher for the working age population. Exploring such heterogeneity in the costs and benefits of the intervention can yield important policy insights for scaling up the intervention. However, it was not feasible to undertake such analyses due to concerns about limited statistical power, particularly when there was no statistically significant difference in the primary outcome (i.e., diabetes incidence). The minimum hourly wage of an unskilled worker employed in the agricultural sector would be an underestimate of opportunity costs for a few participants who were working in higher-level jobs. We used the GDP per capita based WHO-CHOICE threshold to determine the cost-effectiveness of our intervention. Although this threshold is widely used in LMICs [62], it has been criticized for ignoring opportunity costs imposed on health systems [62, 63]. However, our intervention remains cost-effective even with the (much lower) India-specific opportunity-based cost-effectiveness thresholds (US$136.7 to US$915.6 per QALY gained for the year 2018), estimated by Woods et al. [63]. Finally, SF-36 is a generic instrument to measure overall HRQoL, and it could be insensitive to particular aspects of certain health conditions [13], which could be better captured by diabetes-specific HRQoL scales [64].

## Conclusion

In this trial-based economic analysis, we have demonstrated that implementing a low-cost peer-support lifestyle intervention in community settings for individuals at high risk of developing diabetes was cost-effective in a low- and middle-income setting. Future work is needed to determine whether this persists over a longer period of time and when the intervention model is taken to scale. A recent scale-up of K-DPP to more than 15,000 trained peer leaders who delivered the program to more than 370,000 people in Kerala has demonstrated very promising results [65]. The findings of this economic evaluation can help underpin the more effective allocation of scarce healthcare resources for community-based lifestyle interventions for preventing diabetes in India, given the country’s ongoing efforts to establish evidence-based HTA to ensure value for money in the health budget [14].

## Supplementary information

**Additional file 1: Table S1.** Unit costs used for calculating direct and indirect costs. **Table S2.** Baseline characteristics of clusters and participants.

## Data Availability

The datasets used and/or analyzed during the current study are available from the corresponding author on reasonable request.

## Abbreviations

2-h PG: 2-h plasma glucose
ADA: American Diabetes Association
CHEERS: Consolidated Health Economic Evaluation Reporting Standards
CHOICE: CHOosing Interventions that are Cost-Effective
FPG: Fasting plasma glucose
GDP: Gross domestic product
GEE: Generalized estimating equations
GLM: Generalized linear models
HRQoL: Health-related quality of life
ICER: Incremental cost-effectiveness ratio
IDPP: Indian Diabetes Prevention Programme
IGT: Impaired glucose tolerance
K-DPP: Kerala Diabetes Prevention Program
LMIC: Low- and middle-income country
LRP: Local resource person
NNT: Number needed to treat
OGTT: Oral glucose tolerance test
QALY: Quality-adjusted life year
RCT: Randomized controlled trial
SF-36: 36-item Short Form
SF-6D: Short Form 6 Dimension
WHO: World Health Organization
WTP: Willingness to pay
