# Supplementary material for: Cost-effectiveness of a lifestyle intervention in high-risk individuals for diabetes in a low- and middle-income setting: Trial-based analysis of the Kerala Diabetes Prevention Program
Source: BMC Med. 2020 Sep 4;18:251. doi: 10.1186/s12916-020-01704-9 (PMC7472582; doi:10.1186/s12916-020-01704-9)
Supplement: Supplementary file 1 — Additional file 1: Table S1. Unit costs used for calculating direct and indirect costs. Table S2. Baseline characteristics of clusters and participants. [file 12916_2020_1704_MOESM1_ESM.docx]

**Additional file 1**

**Table S1. Unit costs used for calculating direct and indirect costs.**

| Items | Unit | Cost (2018 US$) | Sources |
| --- | --- | --- | --- |
| **Direct medical costs** | | | |
| Personnel  Intervention manager  Intervention assistant  Local resource person  Experts | 1 hour  1 hour  1 hour  1 session | 2.8  1.1  1.6  27.6 | K-DPP accounts register  K-DPP accounts register  K-DPP accounts register  K-DPP accounts register |
| Materials  IDRS  OGTT  Peer leader handbook  Participant workbook  Participant handbook  Health education booklet | 1 unit  1 unit  1 unit  1 unit  1 unit  1 unit | 0.04  4.0  15.3  1.6  1.8  0.8 | K-DPP accounts register  K-DPP accounts register  K-DPP accounts register  K-DPP accounts register  K-DPP accounts register  K-DPP accounts register |
| Healthcare services^*^  Outpatient visits  Public facility  Health centre without beds  Health centre with beds  Primary level hospital  Private facility  Health centre with beds  Primary level hospital  Inpatient days  Public facility  Primary level hospital  Secondary level hospital  Teaching hospital  Private facility  Primary level hospital  Secondary level hospital  Teaching hospital  Medications and diagnostics | 1 visit  1 visit  1 visit  1 visit  1 visit  1 day  1 day  1 day  1 day  1 day  1 day  -- | 2.5  3.2  3.6  4.4  5.1  17.3  18.0  23.3  22.3  23.2  30.0  -- | WHO-CHOICE estimates for India  WHO-CHOICE estimates for India  WHO-CHOICE estimates for India  WHO-CHOICE estimates for India  WHO-CHOICE estimates for India    WHO-CHOICE estimates for India  WHO-CHOICE estimates for India  WHO-CHOICE estimates for India  WHO-CHOICE estimates for India  WHO-CHOICE estimates for India  WHO-CHOICE estimates for India  K-DPP participant self-report questionnaire |
| **Non-medical costs** | | | |
| Transport, food and accommodation costs while seeking health care | -- | -- | K-DPP participant self-report questionnaire |
| Travelling time to attend group sessions | 30 minutes/session | 0.25 | Ministry of Labour and Employment, Govt. of India |
| Time spent during group sessions | 60 minutes/session | 0.5 | Ministry of Labour and Employment, Govt. of India |
| **Indirect costs**  Time lost from paid work due to illness | 1 day | 4.9 | Ministry of Labour and Employment, Govt. of India |

IDRS, Indian Diabetes Risk Score; OGTT, oral glucose tolerance test; K-DPP, Kerala Diabetes Prevention Program; WHO-CHOICE (World Health Organization CHOosing Interventions that are Cost-Effective). ^*^Unit cost for outpatient visits and inpatient days includes personnel, capital and food costs.

**Table S2. Baseline characteristics of clusters and participants.**

| Excluded=250:  Not able to speak, read and write the local language=40  Known T2DM=160  Other chronic illness=39  Pregnancy=2  Taking medications known to influence glucose tolerance=9 | Control group (N=507) | Intervention group (N=500) |
| --- | --- | --- |
| *Cluster level* | | |
| No. of polling areas | 30 | 30 |
| Average no. of individuals per polling area | 1100 | 1150 |
| *Participant level* | | |
| *Socio-demographics* | | |
| Age (years), mean (SD) | 45.7 (7.4) | 46.2 (7.6) |
| Female, n (%) | 236 (46.6) | 239 (47.8) |
| Education, n (%)  Up to primary  Middle  Secondary  Higher secondary  Vocational education  College or above | 117 (23.1)  143 (28.2)  123 (23.5)  42 (8.3)  31 (6.1)  51 (10.1) | 136 (27.2)  129 (25.8)  114 (22.8)  43 (8.6)  28 (5.6)  50 (10.0) |
| Occupation, n (%)  Skilled/unskilled  Homemaker  Unemployed/retired | 361 (71.2)  139 (27.4)  7 (1.4) | 367 (73.4)  129 (25.8)  4 (0.8) |
| Marital status, n (%)  Married  Separated  Divorced  Widowed  Single | 482 (95.1)  2 (0.4)  1 (0.2)  19 (3.8)  3 (0.6) | 476 (95.2)  4 (0.8)  3 (0.6)  9 (1.8)  8 (1.6) |
| Monthly household expenditure (INR), median (IQR) | 6000 (5000 to 10000) | 7000 (5000 to 10000) |
| *Behavioral characteristics* | | |
| ≥5 servings of fruit and vegetables/day^*^, n (%) | 84 (16.6) | 91 (18.2) |
| Physically active (leisure time)^†^, n (%) | 102 (20.4) | 107 (21.1) |
| Current tobacco use^‡^, n (%) | 92 (18.2) | 102 (20.4) |
| Current alcohol use^§^, n (%) | 97 (19.1) | 114 (22.8) |
| Standard drinks of alcohol (on a drinking occasion)^\|\|^, mean (SD) | 0.2 (0.4) | 0.2 (0.4) |
| *Clinical and biochemical characteristics* | | |
| Weight (kg), mean (SD) | 64.5 (12.1) | 62.6 (11.6) |
| Waist circumference (cm), mean (SD) | 88.7 (9.7) | 87.9 (9.7) |
| Waist-to-hip ratio, mean (SD) | 0.93 (0.06) | 0.93 (0.07) |
| Fat percent (%), mean (SD) | 30.0 (8.7) | 29.7 (8.2) |
| Systolic blood pressure (mmHg), mean (SD) | 123.4 (17.9) | 123.0 (17.6) |
| Diastolic blood pressure (mmHg), mean (SD) | 74.8 (12.1) | 75.0 (11.5) |
| IDRS score, mean ± SD | 67.5 (8.4) | 66.8 (8.3) |
| Fasting plasma glucose (mmol/l), mean (SD) | 5.8 (0.5) | 5.8 (0.5) |
| 2-hr plasma glucose (mmol/l), mean (SD) | 6.0 (1.5) | 5.9 (1.6) |
| HbA1c (%), mean (SD) | 5.6 (0.5) | 5.6 (0.5) |
| HbA1c (mmol/mol), mean (SD) | 38 (5.5) | 38 (5.5) |
| Total cholesterol (mmol/l), mean (SD) | 5.7 (1.1) | 5.7 (1.0) |
| LDL cholesterol (mmol/l), mean (SD) | 3.8 (0.9) | 3.8 (0.9) |
| HDL cholesterol (mmol/l), mean (SD) | 1.3 (0.4) | 1.3 (0.4) |
| Triglycerides (mmol/l), median (IQR) | 1.1 (0.9 to 1.6) | 1.2 (0.9 to 1.6) |
| *Medical history* | | |
| Anti-hypertensive drugs, n (%) | 40 (7.9) | 35 (7.0) |
| Lipid-lowering drugs, n (%) | 12 (2.4) | 9 (1.8) |
| *Health-related quality of life (HRQoL)* |  |  |
| Physical component summary of HRQoL scale, mean (SD) | 49.9 (8.5) | 49.1 (8.7) |
| Mental component summary of HRQoL scale, mean (SD) | 53.2 (9.1) | 54.0 (9.1) |
| SF-6D, mean (SD) | 0.78 (0.15) | 0.78 (0.15) |

SD, standard deviation; IQR, inter-quartile range; IDRS, Indian Diabetes Risk Score; SF-6D, Short Form 6 Dimension. Percentages may not add up to 100% due to rounding. ^*^One serving of fruit equals to a medium size fruit or two small size fruits or ½ glass of fruit juice or a bowel of grapes. One serving of vegetables (excludes tubers) equals to 80 grams. ^†^Self-reported history of moderate or vigorous physical activities during leisure time performed in bouts of at least 10 mins duration. ^‡^Smoking or use of smokeless tobacco products (chewing tobacco and snuff) in the past 30 days. ^§^Drank an alcoholic drink in the past 30 days. ^||^One standard drink of alcohol refers to 30 ml of spirits, 120 ml of wine, 285 ml of beer or 285 ml of toddy (palm wine).
